# Novel *POMT2* variants associated with limb-girdle muscular dystrophy R14: genetic, histological and functional studies

**DOI:** 10.1186/s13023-025-03578-7

**Published:** 2025-03-03

**Authors:** Guiguan Yang, Xiaoqing Lv, Wenjing Wu, Guangyu Wang, Mengqi Yang, Yifei Feng, Chuanzhu Yan, Meirong Liu, Pengfei Lin

**Affiliations:** 1 Department of Neurology, Shandong Key Laboratory of Mitochondrial Medicine and Rare Diseases, Research Institute of Neuromuscular and Neurodegenerative Diseases, Qilu Hospital of Shandong University, Shandong University, Jinan, 250012 Shandong China; 2 Department of Neurology, The First Affiliated Hospital of Soochow University, 899 Pinghai Road, Suzhou, 215006 Jiangsu China

**Keywords:** LGMDR14, *POMT2*, Dystroglycanopathies, Genotype-phenotype correlations, Molecular dynamics simulations

## Abstract

**Background:**

The *POMT2* gene, which encodes protein O-mannosyltransferase 2, is essential for α-dystroglycan glycosylation. Variants in *POMT2* cause various disorders, including the relatively rare presentation of limb-girdle muscular dystrophy R14 (LGMDR14).

**Methods:**

This study retrospectively analyzed the clinical, pathological, and genetic data of three LGMDR14 patients. And we investigated the pathogenic mechanisms of *POMT2* variants through aberrant mRNA processing analysis and molecular dynamics simulations to assess their impact on protein structure and function.

**Results:**

We recruited three LGMDR14 patients from unrelated Chinese families, all presenting with adult-onset proximal muscle weakness. All of these patients showed a myopathic pattern on electromyography and decreased α-dystroglycan expression on muscle biopsy. One patient had severe cardiomyopathy and mild cognitive impairment. Genetic sequencing revealed compound heterozygous variants in the *POMT2* gene in all three patients: c.1006 + 1G > A and c.295 C > T in patient 1, c.1261 C > T and c.700_701insCT in patient 2, and c.812 C > T and c.170G > A in patient 3. Variants c.700_701insCT, c.812 C > T, and c.170G > A are novel. Splicing and cDNA analysis revealed that the c.1006 + 1G > A variant could cause retention of the first 26 bp of intron 8 by inducing recognition of new donor splice sites. Pyrosequencing revealed that both frameshift variant c.700_701insCT and splicing variant c.1006 + 1G > A triggered a nonsense-mediated mRNA decay. Molecular dynamics indicated that c.1006 + 1G > A, c.700_701insCT, and c.170G > A variants could lead to truncated proteins, altering stability and function.

**Conclusions:**

Our study summarizes the clinical, pathological and genetic characteristics of three adult-onset LGMDR14 patients, expanding the genetic spectrum of *POMT2* variants. Moreover, the finding reinforces the impact of *POMT2* splicing defects on mRNA regulation, and molecular dynamics simulations predict the structural consequences of *POMT2* variants, providing additional evidence for their functional effects.

**Supplementary Information:**

The online version contains supplementary material available at 10.1186/s13023-025-03578-7.

## Introduction

Alpha-dystroglycanopathies (α-DGPs) include a spectrum of autosomal recessive muscular dystrophies that are characterized by defects in the O-mannosylglycosylation of α-dystroglycan (α-DG) [1, 2]. Alpha-DG connects the extracellular matrix and intracellular actin skeleton, and is important for muscle and nervous system activity [3]. Deficiency of α-DG glycosylation not only impair muscle function but are also implicated in brain and eye development. The proper glycosylation of α-DG is critical for its interaction with extracellular matrix proteins like laminin, which is essential for the correct positioning of neurons during development and for maintaining the structural integrity of retinal tissues [1, 3]. Impaired laminin binding due to defective α-DG glycosylation can lead to neuronal migration defects, causing severe brain malformations such as cobblestone lissencephaly and retinal abnormalities that result in visual impairment [3–5].

Seventeen pathogenic genes are responsible for α-DGP, one of which is *POMT2* (Protein O-Mannosyltransferase 2) gene [4]. The *POMT2* gene, located on chromosome 14q24.3, spans approximately 64 kb and comprises 21 exons. It encodes the 750-amino acid protein O-mannosyltransferase 2 (pomt2), which is primarily expressed in brain and testis tissues [5]. It forms a complex with pomt1, another O-mannosyltransferase, catalyzing the transfer of mannose from dolichol phosphate-mannose to serine or threonine residues of target proteins, such as α-DG. The complex, located in the endoplasmic reticulum, is pivotal for the O-mannosylation pathway, which is indispensable for proper muscle function and neuronal migration during development [5, 6].

Currently, *POMT2*-related disorders include Walker-Warburg syndrome (WWS), congenital muscular dystrophy (CMD) and limb girdle muscular dystrophy type R14 (LGMDR14) [7]. Among these, LGMDR14 is a less common phenotype, and patients typically present with a milder clinical presentation compared to those with CMD and WWS. LGMDR14 typically manifests during childhood or adolescence, with initial symptoms of proximal limb muscle weakness and atrophy [4, 6, 7]. Some patients also present with mild cognitive impairment and cardiac involvement. Diagnosis primarily relies on clinical presentation, muscle biopsy findings, and genetic testing. There is currently no effective treatment for LGMDR14 [7, 8].

The first LGMDR14 patient was reported in 2007. She presented with proximal muscular dystrophy without brain involvement and severe α-DG reduction on muscle pathology [9]. Up to now, 44 *POMT2* variants have been identified in LGMDR14 patients, and most of them belongs to missense variants (75%) [10]. Few intronic or frameshift variants were observed. In China, only four LGMDR14 cases have been reported [4, 8]. According to the HGMD database, 17 intronic variants have been identified in the *POMT2* gene, but the specific splicing mechanism of these variants remains largely unexplored. Molecular dynamics simulation to analyze atomic and molecular physical movements has not been widely applied in *POMT2*-related disease studies, despite its potential to elucidate the structural and functional consequences of variants [11, 12]. This method contributes to understanding of the molecular basis of genetic disorders associated with variants in *POMT2* [13].

This study summarizes the clinical, histological, and genetic findings in three adult-onset LGMDR14 patients, highlighting novel variants in the *POMT2* gene. Molecular genetic analysis was conducted to investigate the effects of identified variants on mRNA processing and nonsense-mediated mRNA decay (NMD) mechanisms. Moreover, we used molecular dynamics (MD) to predict the effects of the *POMT2* variants on the protein structure and function. The pyrosequencing findings, along with the identification of novel splicing and NMD events, broaden the spectrum of variants associated with *POMT2* and enhance its diagnostic utility for LGMDR14.

## Methods

### Subjects and clinical assessments

Patients from three Chinese families were diagnosed with LGMDR14 at Qilu Hospital of Shandong University between 2015 and 2023. We conducted a retrospective analysis of the clinical data, including onset age, primary symptoms, family history, and physical and auxiliary examinations. Muscle strength was evaluated using the Medical Research Council (MRC) scale.

### Muscle pathology

Diagnostic muscle samples were procured from the left quadriceps femoris of patient 1 and the right quadriceps femoris of patients 2 and 3. These specimens were promptly cryopreserved in liquid nitrogen and maintained at − 80 °C. We conducted standard histological and histochemical procedures, including hematoxylin and eosin (H&E) staining, modified gomori trichrome (MGT) staining, nicotinamide adenine dinucleotide tetrazolium reductase (NADH-TR) and ATPase (pH = 4.3/10.4) staining. The following primary antibodies were utilized for immunohistochemical evaluation with standard procedures: dystrophin (DYS1: rod domain, DYS2: C-terminus, and DYS3: N-terminus; Novocastra, Newcastle, UK), dysferlin (NCL-Hamlet; Novocastra, Newcastle, UK), and α-DG, IIH6C4 (Millipore, US). Control muscle specimens from patients with mild myopathic changes were used to conduct a comparative analysis. Informed consent was obtained from the patients in research and publication of related findings.

### Whole exome sequencing and Sanger sequencing

Genomic DNA was isolated from peripheral blood samples of affected individuals, their relatives, and healthy controls following the manufacturer’s protocol. Whole exome sequencing (WES) was performed on three patients to identify potential causative variants [14, 15]. Extracted DNA underwent fragmentation and purification, followed by adaptor ligation and target enrichment using custom-designed NimbleGen SeqCap probes (Roche NimbleGen). Indexed libraries were sequenced on a NextSeq500 platform (Illumina). Sequencing reads were aligned to the human reference genome (GRCh37/hg19), and variant calling was performed using NextGENe v2.4.1.2 software (SoftGenetics) [16]. Identified variants were annotated with information from population and disease databases [16]. Variants from the proband were analyzed collectively, with annotation of their source. Synonymous variants and common single nucleotide polymorphisms (SNPs) with a minor allele frequency (MAF) greater than 0.1% were filtered out. Rare, high-confidence variants were considered as potential disease-causing candidates. Candidate variants pathogenicity were assessed using in silico algorithms (SIFT, PolyPhen-2, CADD) and population databases (OMIM, dbSNP, gnomAD, ClinVar). Variant interpretation followed the American College of Medical Genetics and Genomics (ACMG) guidelines [16, 17].

Sanger sequencing was employed to validate the variants identified through WES in the proband and their parents. PCR primers targeting the candidate loci were designed based on the human reference genome sequence (GRCh37/hg19) obtained from GenBank in NCBI. Polymerase chain reaction (PCR) amplification of the target regions was performed, followed by direct sequencing of the PCR products using a 3500XL Genetic Analyzer (Applied Biosystems, USA) [14, 15]. Primers for PCR amplification were designed using Primer Premier 5.0 software. The primer sequences are provided in the Supplementary Materials.

### cDNA analysis

We used TRIzol reagent to extract the total RNA from muscle biopsies of patients 1 and 2 and performed reverse-transcription using HiScript III RT SuperMix for qPCR (+ gDNA wiper) (Vazyme, China) to obtain cDNA. To detect the aberrant splicing of *POMT2* mRNA, a pair of primers was designed for reverse-transcription PCR (RT-PCR). The forward primer was 5’-GCTGTTCACTTCATGGTGC-3’, and the reverse primer was 5’-GTGATCACAGAGCCGTAGG-3’. The PCR product, which covered exons 7–9, was 137 bp in length. We isolated the amplified fragment using DNA agarose gel electrophoresis, cloned it into the pMD18-T vector and transformed it into *E. coli* DH5α. Then, we performed colony PCR and sequencing to identify the abnormal *POMT2* mRNA splicing mechanism.

### Pyrophosphate sequencing and quantitative PCR assay

Pyrosequencing is a technique to quantitatively analyze of genetic variant abundance by measuring the amount of pyrophosphate (PPi) released during cDNA synthesis. We conducted pyrosequencing to analyze the relative amount of mutant transcripts in patients 1 and 2. For the polymerase chain reaction (PCR) in patient 1, the forward primer was 5’-CGCACATCTGTTGGGATGA-3’, and the reverse primer was 5’-CAGGTAGCCAGCAAGACCTATC-3’. The pyrosequencing primer used was 5’-AATGGGAAGTTACTATATCA-3’. For the polymerase chain reaction (PCR) in patient 2, the forward primer was 5’-ACCCTTCCTTCCCAGTGGAGTT-3’, and the reverse primer was 5’-CTGATAGTGCTTCCGGGTCATG-3’. The pyrosequencing primer used was 5’-CTAGAACACAAAGAAACTTC-3’. Pyrosequencing was conducted using the PyroMark Q96 ID platform (QIAGEN, Germany). Subsequent analysis of single nucleotide polymorphisms (SNPs) at each locus was automatically performed with the Pyro Q-CG software. We also performed a quantitative real-time PCR (qPCR) assay to detect the *POMT2* mRNA levels in the patients and controls, and a detailed methodology is provided in the Supplementary Materials.

### Computational structural modeling of the *POMT2* variants

The human pomt2 protein sequence was retrieved from UniProt (ID: Q9UKY4). Predictive 3D models were generated using AlphaFold 2.3 and visualized via PyMOL (v2.0.6). Structural and interaction alterations at variant sites were analyzed. The Imutant 2.0 software was used to analyze the effect of the missense variant c.812 C > T on the structural stability and calculate the free energy (ΔΔG) of the protein.

### Molecular dynamics

Molecular dynamics (MD) simulations were executed using GROMACS 2020.3 based on the derived variant 3D models [11, 12]. Small molecule preprocessing involved AmberTools22 for GAFF force field application and Gaussian 16 W for hydrogenation and RESP potential calculations. The calculated potential data were incorporated into the topology file of the molecular dynamics system. The simulation conditions were conducted at a constant temperature of 300 K and atmospheric pressure of 1 bar, employing the Amber99sb-ildn force field, with the Tip3p water model as the solvent. System charge neutrality was achieved by adding NaCl ions as needed. The MD simulation protocol comprised energy minimization, isothermal isovolumic ensemble (NVT) and isothermal isobaric ensemble (NPT) equilibration, and production dynamics. Energy minimization utilized the steepest descent method, followed by 100,000 steps of NVT and NPT equilibration, each with a 0.1 ps coupling constant and 100 ps duration. The production phase involved 5,000,000 steps with a 2 fs step size, totaling 100 ns. After the simulation, the relevant parameters were carefully analyzed using the GROMACS software package [18]. Detailed calculations and statistical analyses of relevant parameters are provided in the Supplementary Material.

## Results

### Clinical findings

#### Case 1

Patient 1 was a 45-year-old female from a non-consanguineous family with no familial neuromuscular disease history. He exhibited normal motor development during childhood. At approximately 25 years of age, she began developing proximal lower limb weakness, which subsequently progressed. Currently, she exhibits an unsteady gait and experiences difficulty performing basic movements such as standing up from squatting, bending down and climbing stairs. The physical examination revealed a decrease in muscle strength in the limbs. Specifically, muscle strength in the upper limbs was graded as 4/4 (MRC scale [left/right]) for the deltoid and biceps brachii. In the lower limbs, the muscle strength was rated as 3-/4 for iliopsoas, 4-/4 for quadriceps femoris, 4-/4 for biceps femoris, 4+/5 for gastrocnemius and tibialis anterior. Laboratory tests revealed an increase in serum creatine kinase (CK) level of 5624 U/L (normal range:38–174 U/L). The ophthalmologic and cognitive assessments were normal. Electromyography (EMG) suggested myogenic impairment, whereas the nerve conduction remained normal. The echocardiogram did not reveal any notable abnormalities in the cardiac muscle. On muscle MRI images, fatty infiltration was predominantly found in the semimembranosus, semitendinosus, and biceps femoris (Fig. S1 A).

#### Case 2

Patient 2 was a 39-year-old male with no familial history of neuromuscular disorders and demonstrated normal motor development in childhood. At approximately 21, he began exhibiting proximal lower limb weakness, which progressively worsened. At 31 years of age, he was unable to squat, walked with waddling and needed assistance in climbing stairs. Four years ago, he presented with progressive heart failure symptoms and was diagnosed with dilated cardiomyopathy. Currently, he has become wheelchair-bound. On physical examination, the patient showed obvious weakness in the lower and upper limb muscles. In the lower extremities, the iliopsoas muscle strength was rated as 2/3, the quadriceps femoris strength was rated as 4/4, the biceps femoris and tibialis anterior strength was rated as 4-/5-, and the gastrocnemius strength was rated as 4/4. In the upper extremities, the triceps brachii and biceps brachii muscle strength was rated as 4/4, and the deltoid strength was rated as 3/4. The bilateral tendon reflexes disappeared. Laboratory examinations revealed elevated CK (1252 U/L) and N-terminal proB-type natriuretic peptide (NT-proBNP: 285.7 pg/mL; normal range: 0–125 pg/mL). The brain MRI was normal, whereas the Mini-Mental State Examination (MMSE) indicated mild cognitive impairment (22/30). EMG indicated myogenic impairment. Echocardiography revealed cardiac muscle abnormalities, which included reduced left ventricular systolic and diastolic function with left atrial enlargement (left ventricular ejection fraction: 43%; normal range: 50-70%) and moderate mitral regurgitation. The electrocardiogram revealed T-wave abnormalities.

#### Case 3

Patient 3 was a 53-year-old woman born to non-consanguineous family with normal motor development. At the age of 43, she presented fatigue after walking and over the course of 5 years, she began to notice gradual proximal muscle weakness in her lower limbs and gait instability. Currently, she experiences difficulty standing from a squat and bending over, and she has intermittent back pain. Neurological examination revealed reduced muscle strength in the proximal leg muscles. In the lower extremities, the iliopsoas muscle strength was rated as 3/2, that of the biceps femoris was rated as 4/3, and that of the quadriceps femoris was rated as 4/5-. The facial and neck muscles were normal. Nerve sensory examination was normal. Laboratory examinations revealed elevated CK level (527 U/L). EMG indicated myogenic impairment. Brain MRI and cognitive development findings were normal. Echocardiography revealed no apparent abnormalities in the cardiac muscle. On muscle MRI results, fatty infiltration was predominantly found in the semimembranosus, semitendinosus, and biceps femoris (Fig. S1 B).

The detailed clinical data of the three patients are presented in Table 1.

**Table 1:** Characteristics of three patients with LGMD R14

	Patient 1	Patient 2	Patient 3
Gender/age(year)	Female/45	Male/39	Female/53
Onset age(year)	25	21	43
Disease Course	20	18	10
Family history	No	No	No
Presenting symptoms	Proximal lower limb weakness and difficulty in climbing stairs and squatting	Proximal lower limb weakness and wheelchair-bound	Proximal lower limb weakness and difficulty in squatting
Muscle strength of upper limbs DT BB TB Distal	4/4 4/4 5/5 5/5	3/4 4/4 4/4 5/5	5/5 5/5 5/5 5/5
Muscle strength of lower limbs IP QF BF TA GC	3- /4 4- /4 4-/4 4+/5 4+/5	2/3 4/4 4-/5- 4-/5- 4/4	3/2 4/5- 4/3 5/5 5/5
Neck muscles	Normal	Normal	Normal
Muscle atrophy	Normal	Thigh muscles	Normal
Joint contracture	Normal	Normal	Normal
Mobility status	Ambulatory	wheelchair-bound	Ambulatory
Brain MRI	Normal	Normal	Normal
Electroencephalogram	Normal	Normal	Normal
Mental retardation	Normal	Mild	Normal
Echocardiogram	Normal	Dilated cardiomyopathy	Normal
Electrocardiogram	Normal	T-wave abnormalities	Normal
Respiratory evaluation	Normal	Normal	Normal
Ocular involvement	Normal	Normal	Not done
Electromyogram	Myogenic change	Myogenic change	Myogenic change
Muscle MRI	Fatty infiltration in bilateral posterior thigh	Not done	Fatty infiltration in bilateral posterior thigh
CK (U/L)	5624	1252	527
LDH (U/L)	869.6	382.5	298.1
Muscle biopsy	Muscular dystrophic change, reduced α-DG expression	Muscular dystrophic change, reduced α-DG expression and RVs	Muscular dystrophic change, reduced α-DG expression
*POMT2* Variant	c.1006 + 1G > A; c.295 C > T (p.R99C)	c.700_701insCT (p.V234Afs*8); c.1261 C > T (p.R421W)	c.812 C > T (p.S271L); c.170G > A (p.W57*)

Abbreviation: IP, iliopsoas; QF, quadriceps femoris; BF, biceps femoris; TA, tibialis anterior; GC, gastrocnemius; BB, biceps brachii; TB, triceps brachii; DT, deltoid; RVs, rimmed vacuoles in muscle pathology

### Histological analysis

**Fig. 1:**
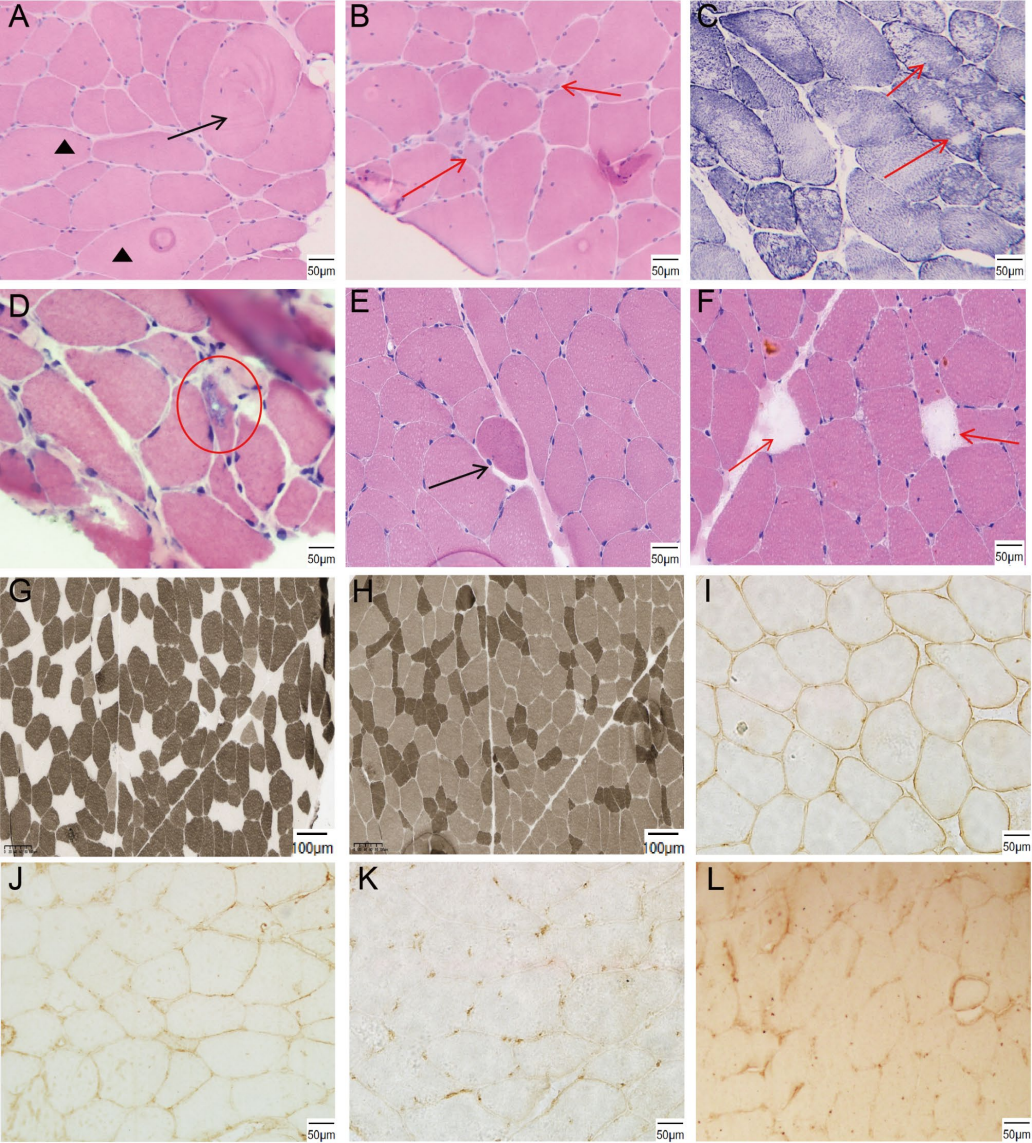
Histological analysis of three LGMDR14 patients. **(A, B, C)** HE and NADH staining of Patient 1. **(A**,** B)** HE staining revealed different sizes of muscle fibers, necrotic and regenerated muscle fibers (red arrows), hypertrophic split muscle fibers (black arrow), an increase in muscle fibers with internal nuclei (black triangle), and mild hyperplasia endomysium. **(C)** NADH staining revealed many moth-eaten muscle fibers (red arrows). **(D)** HE staining revealed various sizes of muscle fibers and basophilia vacuoles in Patient 2 (red circle). **(E**,** F**,** G**,** H)** Histological analysis of Patient 3. **(E**,** F)** HE staining revealed various sizes of muscle fibers, internal nuclei, highly-contractile fibers (black arrow) and necrotic muscle fibers (red arrows). **(G)** ATPase (pH = 4.3) staining revealed a preponderance of type I fibers. **(H)** ATPase (pH = 10.4) staining revealed atrophy in size of type II fibers. **(I**,** J**,** K**,** L)** Immunohistochemical staining analysis of α-DG in the control **(I)**, patient 1 **(J)**, patient 2 **(K)** and patient 3 **(L)**. Patients presented significantly reduced α-DG expression in their sarcolemma compared with the control group

Muscle pathology revealed myogenic changes in all three patients. Histological analysis revealed many common characteristics, including fiber size variation, an internal nuclei increasement, regenerating fibers and occasional necrosis fibers (Fig. 1A-F). In addition, Patient 1 exhibited hypertrophic and split muscle fibers in H&E staining and moth-eaten muscle fibers in NADH staining (Fig. 1A, C). Muscle biopsy revealed rimmed vacuoles in patient 2 (Fig. 1D). Patient 3 presented highly contractile fibers, type I fibers preponderance and type II fibers atrophy in ATPase staining (Fig. 1E-H). Immunohistochemical staining revealed a significant decrease in the expression of α-DG in the sarcolemma of the three patients compared with the control group. (Fig. 1I-L). However, other proteins, such as dystrophin-N, dystrophin-R, dystrophin-C, and dysferlin, did not reveal abnormalities.

### Genetic analysis

**Fig. 2:**
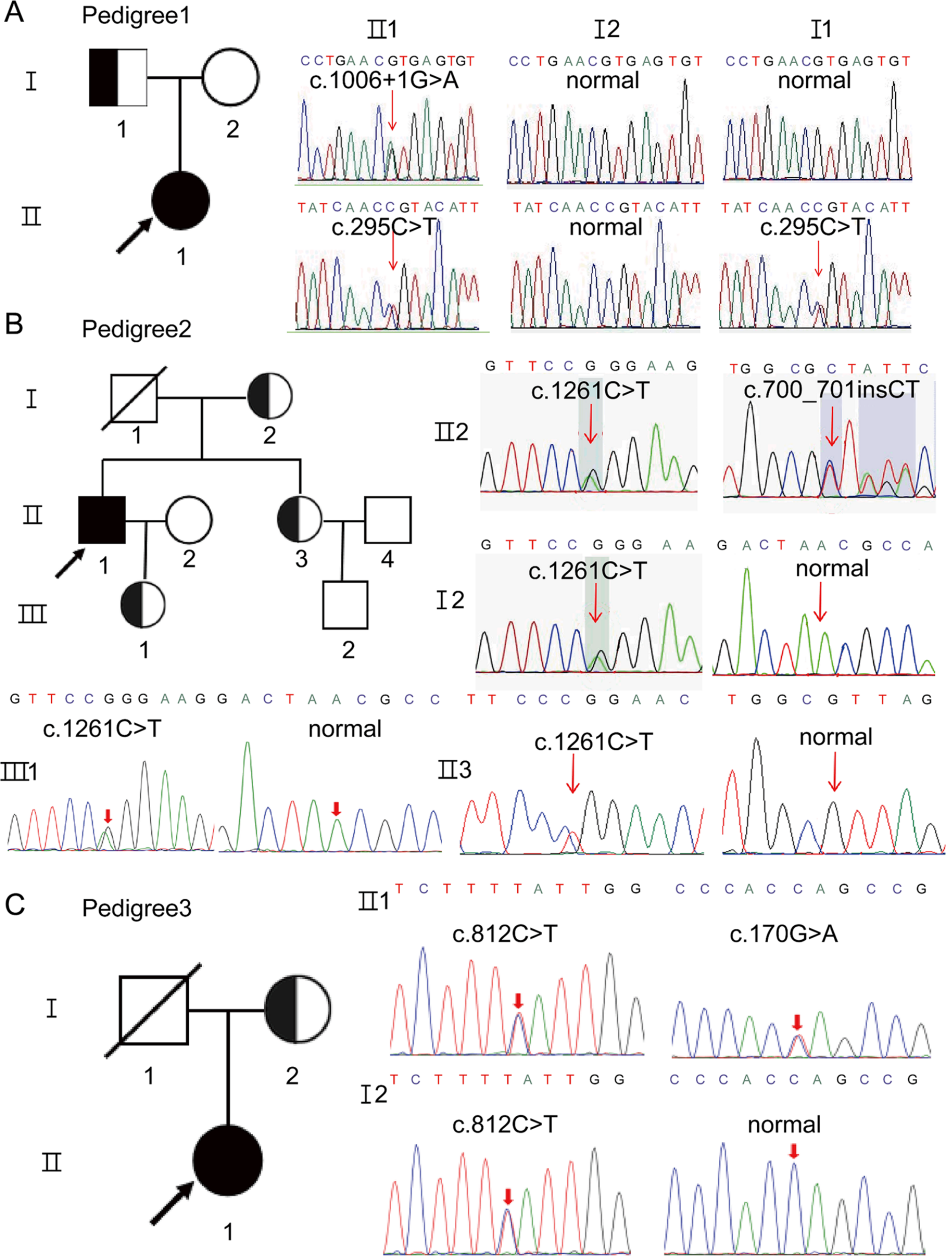
Pedigree analysis of three patients and Sanger analysis. **(A)** Pedigree of Patient (1) c.1006 + 1G > A and c.295 C > T compound heterozygous variants were identified in Patient 1, and her father (I1) carried the c.295 C > T heterozygous variant. **(B)** Pedigree of Patient (2) c.700_701insCT and c.1261 C > T compound heterozygous variants were identified in Patient 2, and his mother (I2), daughter (III1) and sister (II3) all carried the c.1261 C > T heterozygous variant. (**C)** Pedigree of Patient (3) c.812 C > T and c.170G > A compound heterozygous variants were identified in Patient 3, and her mother (I2) carried the c.812 C > T heterozygous variant. Variants in the *POMT2* were indicated by red arrows

**Fig. 3:**
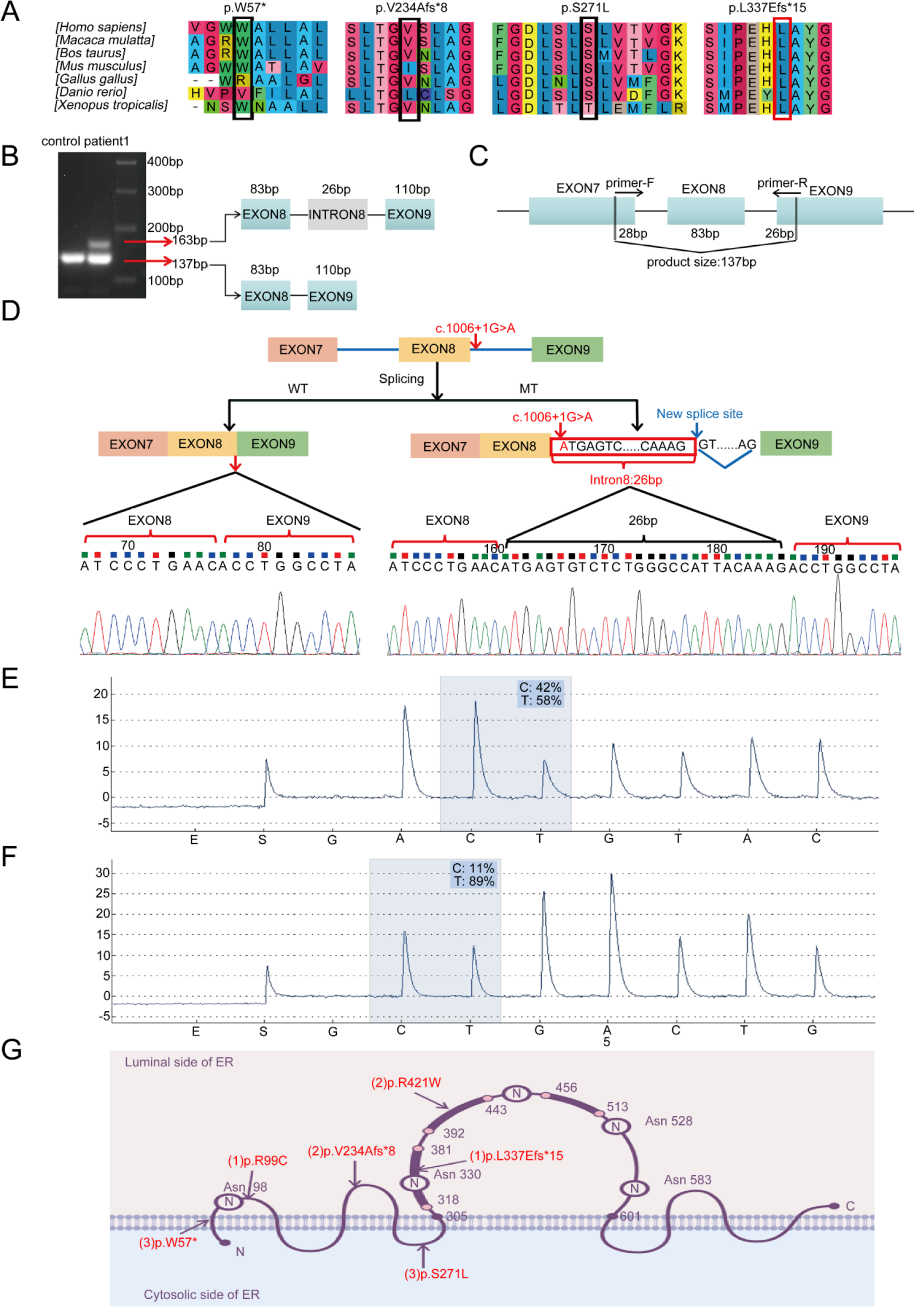
cDNA and splicing analysis. **(A)** Sequence alignment analysis revealed a significant conservation of the amino acid residues p.W57, p.V234, p.S271, and p.L337 across various species. (**B)** The agarose gel electrophoresis indicated that the band denoting the standard size level was significantly thicker than the band denoting the enlarged size. (**C)** A set of primers was specifically designed for Patient 1, resulting in a 137 bp PCR fragment that covered exons 7 to 9. (**D)** The schematic diagram illustrated the impact of the variation, with two different transcripts generated through WT (Wild-Type) and MT (Mutation) splicing. The c.1006 + 1G > A variant was found to induce the retention of the first 26 bp of intron 8. (**E)** Pyrosequencing analysis revealed that 42% of the transcripts from Patient 1 carried the c.1006 + 1G > A variant, with the remaining 58% lacking the variant. (**F)** Pyrosequencing analysis revealed that only 11% of the transcripts from Patient 2 had the c.700_701insCT variant, whereas the remaining 89% lacked the variant. (**G)** The schematic diagram of pomt2 protein and the distribution of variants in three patients

We considered the diagnosis of LGMD in these three patients based on their clinical and pathological findings. Genetic analysis through WES identified several variants in the *POMT2* gene. In this study, the *POMT2* gene sequence corresponds to NM_013382.7, and the protein sequence for pomt2 encoded by *POMT2* is denoted as NP_037514.2. Patient 1 carried the compound heterozygous c.1006 + 1G > A and c.295 C > T (p.R99C) variants in the *POMT2* gene (Fig. 2A). Her father carried the c.295 C > T variant and her mother carried no variant. Patient 2 carried the compound heterozygous c.700_701insCT (p. V234Afs*8) and c.1261 C > T (p.R421W) variants (Fig. 2B). The mother, sister and daughter of the patient carried the c.1261 C > T variant. Patient 3 carried the compound heterozygous c.812 C > T (p.S271L) and c.170G > A (p.W57*) variants, and her mother carried the c.812 C > T variant (Fig. 2C). The fathers of patients 2 and 3 were died, precluding genetic verification. The frequency of the six variants was 0 in the ExAC, gnomAD and TGP databases. Notably, c.1006 + 1G > A was reported in WWS but not in LGMDR14 [19, 20]. The novel variants c.700_701insCT, c.812 C > T and c.170G > A have never been reported in the HGMD and ExAC databases. Bioinformatic tools such as SIFT, CADD and MutationTaster, predict that these variants are deleterious. In addition, conservation analysis using ClustalW2 confirmed the high conservation of the affected amino acids across species (Fig. 3A and Fig. S2). According to the American College of Medical Genetics and Genomics (ACMG) guidelines [21], the c.700_701insCT variant was rated as likely pathogenic (PM2_Supporting + PVS1), the c.812 C > T variant was rated as likely pathogenic (PM2 + PM3 + PP3 + PP4), and the c.170G > A variant was rated as likely pathogenic (PM2_Supporting + PVS1). Therefore, we concluded that the three novel variants were deleterious.

### Splicing and cDNA analysis

DNA electrophoresis of cDNA of patient 1 revealed two distinct bands: one at 137 bp and the other at 163 bp. Notably, the 137-bp band exhibited a slightly greater intensity than the 163-bp band. In contrast, healthy individual displayed only a single band at 137 bp (Fig. 3B). These results suggested that the aberrant splicing of *POMT2* mRNA was caused by the canonical + 1 splice site variant. After the PCR products had been purified via gel extraction and transformed into *E. coli* DH5α, the colony PCR and sequencing revealed that the *POMT2* c.1006 + 1G > A variant induced intron retention (IR), which indicated that the first 26 bp of intron 8 were retained by the disruption of the canonical donor splice site and induced the recognition of cryptic donor splice sites at 26 bp downstream (Fig. 3B-D). At the protein level, the retention of 26 bp induced p. L337Efs*15 according to the ExPASy translation tools (https://web.ExPASy.org/translate/). The leucine at position 337 is highly conserved across mammalian species (Fig. 3A).

### Pyrophosphate sequencing and quantitative PCR assay

Pyrosequencing revealed that transcripts harboring the c.1006 + 1G > A variant comprised 42% of the total, whereas transcripts lacking the variant comprised the remaining 58% (Fig. 3E). A quantitative PCR assay reveal that 22% of the *POMT2* mRNA was degraded in the quadriceps femoris of the Patient 1 compared with the control (Fig. S3A). Since the variant c.1006 + 1G > A was not near the final exon and the stop codon occurred before the theoretical nonsense-mediated mRNA decay (NMD) boundary, NMD was likely triggered. The results obtained from pyrosequencing, qPCR, and agarose gel electrophoresis (Fig. 3B) revealed that transcripts harboring the c.1006 + 1G > A variant underwent partial degradation. This finding suggested that this alternative transcript triggered a partial NMD response. Patient 2 exhibited a frameshift at the protein level due to the c.700_701insCT variant ( p. V234Afs*8). Pyrosequencing analysis indicated that transcripts containing the c.700_701insCT variant constituted only 11% of the total, and the remaining 89% were variant-free transcripts (Fig. 3F). Additionally, a quantitative PCR assay revealed that 46% of the *POMT2* mRNA was degraded in the quadriceps femoris of the Patient 2 compared with the control (Fig. S3B). These results strongly suggested that most transcripts harboring the c.700_701insCT variant were targeted for degradation by the NMD pathway. Consequently, a substantial decrease in the production of mutant protein was anticipated due to the degradation of the mutant mRNA transcripts by NMD.

### 3D model analysis of the abnormal structure of pomt2 caused by variants

**Fig. 4:**
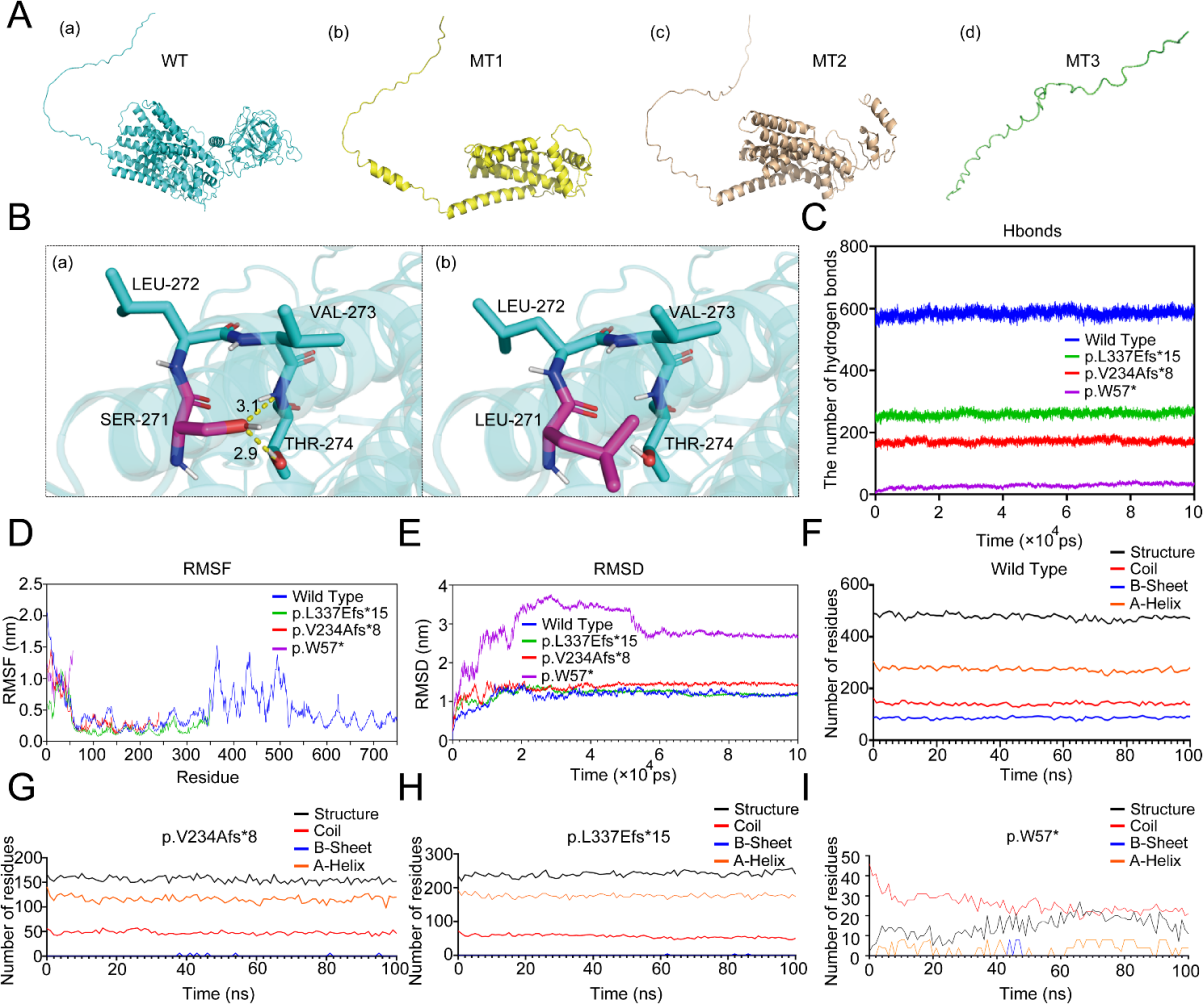
3D model analysis and protein molecular dynamics simulation of the four protein structures (WT, MT1, MT2 and MT3). **(A)** The 3D model of pomt2 structure that was predicted through Alphafold2. (a) The wild-type pomt2 structure. (b) MT1: The truncated structure of p.V234Afs*8. (c) MT2: The truncated structure of p.L337Efs*15. (d) MT3: The truncated structure of p.W57*. **(B)** The c.812 C > T (p.S271L) variant caused the 271th amino acid and its surrounding side chain to shift, resulting in the loss of two hydrogen bonds between SER271 and THR274. **(C)** The number of hydrogen bonds of the WT and three MT chains during 100 ns. **(D)** RMSF of each amino acid of the WT and three MT chains. **(E)** RMSD of the WT and three MT chains during 100 ns. **(F)** The number of the main secondary structures of WT chain. **(G)** The number of the main secondary structures of MT1 chain. **(H)** The number of the main secondary structures of MT2 chain. **(I)** The number of the main secondary structures of MT3 chain

The novel c.812 C > T (p.S271L) variant in the *POMT2* gene resulted in the substitution of serine (Ser) at position 271 with leucine (Leu). This variant occurred at the junction loop between two alpha-helices of the protein. The variant to Leu altered the side chain from CH2OH to CH2CH(CH3)2 and increased its size. Ser is a polar amino acid, whereas Leu is hydrophobic. The pKa values were 5.68 for Ser and 5.98 for LEU, which indicated a slight increase in protein alkalinity. The variant also shifted the 271st amino acid and its surrounding side chain, which resulted in the loss of two hydrogen bonds between SER271 and THR274 (Fig. 4B). Imutant 2.0 showed that the variant changed the free energy of the pomt2 protein (ΔΔG= -1.34 kcal/mol < 0), which decreased the protein stability.

At the protein level, all variants c.1006 + 1G > A, c.700_701insCT and c.170G > A resulted in a truncated protein production in p. L337Efs*15 (350 amino acids), p.V234Afs*8 (240 amino acids) and p.W57* (56 amino acids), respectively. We used homology modeling to generate the 3D structure of three truncated proteins and the wild-type protein (Fig. 4A). According to the schematic diagram of pomt2 [22] (Fig. 3G), pomt2 has nine transmembrane domains that catalyze O-mannosylation. The enzyme activity of pomt2 is influenced by five N-glycosylation sites, all of which are located on the luminal side of the endoplasmic reticulum [22]. The final four sites can be found within a sizable hydrophilic loop encompassing amino acids 305–601, which are essential to the enzyme’s function. In this loop, there are three presumed homologous domains known as the MIR domains, which are crucial for the catalytic activity of pomt2 [23]. After the protein truncation, these effects cause the loss of the hydrophilic loop structure and some N-glycosylation sites. Therefore, these changes can affect the normal glycosylation process of pomt2 and are likely pathogenic based on their position and effect.

### Molecular dynamics simulation of the pomt2 protein in the wild-type strain and three truncated pomt2 strains

To investigate the differences in geometric structure between the wild-type (WT) and three mutant (MT) pomt2 chains (MT: MT1: p. V234Afs*8, MT2: p. L337Efs*15, MT3: p.W57*), we used molecular dynamics simulations over a 100-ns timescale. RMSD analysis revealed that while WT, MT1, and MT2 reached equilibrium within 20 ns, MT3 required significantly longer, demonstrating its inherent instability (Fig. 4E and Fig. S4). Notably, MT1 exhibited increased RMSD compared to WT, indicating reduced structural stability, while MT3 showed markedly greater RMSD values and volatility, highlighting its profound instability. RMSF analysis revealed altered N-terminal (0–50 residues) dynamics in MT1 and MT2 compared to WT, with MT3 displaying drastically increased fluctuations, further emphasizing its destabilization. Furthermore, a decrease in SASA and Rg across all mutants suggested reduced flexibility and impaired interaction potential (Fig. S4).

Hydrogen bond analysis unveiled a significant reduction in hydrogen bonds for MT1, MT2, and MT3 compared to WT, indicating compromised ligand binding affinity (Fig. 4C and Fig. S4). These findings indicated disrupted catalytic and binding function in the mutants. Secondary structures analysis revealed that the number of coils and α-helices in the MT chains significantly decreased compared with those in the WT chains, and the β-sheet structure was almost completely lost (Fig. 4F-I and Fig. S4). A reduction in these major secondary structures would further contribute to their reduced stability and altered interaction capabilities. These results provided novel insights into the structural and dynamic consequences of *POMT2* variants, highlighting their disruptive impact on protein stability, ligand binding, and secondary structure integrity.

## Discussion

LGMDR14, which is a rare autosomal recessive subtype of LGMD, is attributed to genetic variants in the *POMT2* gene [4]. Here, we summarized the clinical and genetic characteristics of three Chinese patients with adult-onset LGMDR14. The muscle weakness presented in adulthood in our three patients. According to Panicucci et al., the median age of onset for LGMDR14 patients was 4 years [10]. However, the onset ages of our patients were all after 20 years old, which suggested that the adult-onset LGMD type also deserved attention. All of these patients had common clinical features, including slowly progressive proximal muscle weakness, elevated CK levels and myogenic changes on EMG. However, patient 2 had severe cardiac involvement and mild cognitive impairment. Cardiac muscle and brain tissues also need α-DG glycosylation, which could explain the common incidence of cardiac and cognitive involvement in LGMDR14 patients [7]. These findings emphasize the importance of regular cardiac and cognitive assessments in LGMDR14 management. In terms of muscle pathology, all three patients presented myogenic damage and a reduction in α-DG, which further confirmed the diagnosis of LGMDR14. In addition, patient 1 exhibited moth-eaten muscle fibers, and patient 3 presented a preponderance of type I fibers and atrophy in type II fibers (Fig. 1), which have never been described in previous reports. These suggested myofibrillar network disorganization and uneven fiber type distribution. Moreover, in this study, we identified three novel variants and studied the splicing and pathogenicity mechanisms of them.

For patient 1, we identified a novel splicing variant c.1006 + 1G > A in *POMT2* and conducted a detailed analysis of the effect of the splicing mechanism. Functional analysis showed that the splicing variant c.1006 + 1G > A induced the retention of the first 26 bp of intron 8 by during mRNA processing (Fig. 3B-D). This occurred through disruption of the canonical donor splice site and induction of recognition of a downstream cryptic donor splice site. In addition to shortened transcripts, transcripts that involve exon skipping and intron retention may also be subject to degradation by NMD [24]. Through pyrosequencing and qPCR, we discovered that the splice variant c.1006 + 1G > A induced partial degradation via nonsense-mediated decay (NMD) (Fig. 3E and Fig. S3). The HGMD database reported 17 intron variants in the *POMT2* gene in total, but few studies elucidated their specific splicing mechanism. Yanagisawa et al. identified two c.248 + 5G > C and c.1333-14G > A intronic variants in the CMD patients [25]. They studied that c.248 + 5G > C led to a 72-bp insertion between exons 1 and 2, and c.1333-14G > A resulted in a 12-bp retention between exons 12 and 13 [25]. In previous researches, the c.1006 + 1G > A variant was confirmed to be associated with WWS and cobblestone lissencephaly in two patients [19, 20]. Reeuwijk et al.firstly identified the c.1006 + 1G > A homozygous variant in a male child who presented with severe WWS [19]. And Devisme et al.reported another patient with cobblestone lissencephaly who carried c.1006 + 1G > A and c.1168_1172delCATA complex heterozygous variants [20]. Compared with Patient 1, both patients had more severe clinical manifestations. These results showed that the same variant site could appear in different phenotypes of *POMT2*-associated diseases, which reinforced the complexity between genotype and phenotype.

Three novel variants, c.700_701insCT, c.812 C > T and c.170G > A, have not previously been reported in *POMT2*-related disorders, which further expanded the genetic spectrum of the *POMT2* gene. The c.700_701insCT was a frameshift variant. At the protein level, the variant c.700_701insCT resulted in p.V234Afs*8. Our results indicated that the majority of transcripts carrying the c.700_701insCT variant underwent NMD degradation (Fig. 3F and Fig. S3). This degradation was expected to significantly reduce the production of the mutant protein. Patient 3 harbored compound heterozygous variants, including c.812 C > T (p.S271L) and c.170G > A (p.W57*). The amino acids at positions p.S271 and p.W57 displayed marked conservation and were anticipated to induce significant damage based on computational predictions (Fig. 3A). In the 3D model, the c.812 C > T (p.S271L) shifted the 271st amino acid and its surrounding side chain, which resulted in the loss of two hydrogen bonds between SER271 and THR274 (Fig. 4B). The nonsense variant c.170G > A (p.W57*) caused the premature appearance of stop codons and formed a truncated protein, which could generate severe consequences for the protein function. Therefore, we concluded that the c.700_701insCT, c.812 C > T and c.170G > A variants were deleterious.

Protein O-Mannosyltransferase 2 is the enzyme that catalyzes the initial step of α-DG O-mannosylation and has been implicated in severe phenotypic manifestations [20]. The earliest documented case of *POMT2* variant was WWS, which exhibited the most severe phenotype [19]. Recent advancements in genetic testing have revealed a spectrum of milder *POMT2*-related phenotypes such as CMD and LGMDR14. However, due to the pleiotropic effects of genes, a definitive connection between genotype and phenotype was not evident, and a range of phenotypes could be induced by identical variant sites in different individuals. Our results validated the hypothesis that the clinical phenotype of *POMT2*-related disorders was influenced by the type and location of the variant [22, 26]. In LGMDR14 patients, the presence of at least one missense variant was common, likely contributing to a residual level of pomt2 enzyme activity that improved the clinical phenotype [25, 26]. Conversely, WWS patients typically harbored two ‘null’ variants, resulting in severe protein dysfunction (such as truncation or alteration of crucial catalytic residues), which exacerbated their clinical phenotype [19]. Given that patient 1 had a point variant (c.295 C > T) associated with minimal protein functional impact and an intron variant (c.1006 + 1G > A) leading to a truncated protein, we hypothesized that patient 1 may present a relatively mild phenotype. Similarly, patients 2 and 3, each harboring a missense variant [c.1261 C > T (p.R421W) and c.812 C > T (p.S271L), respectively] paired with a null variant [c.700_701insCT (p.V234Afs*8) and c.170G > A (p.W57*), respectively], also presented with milder phenotypes relative to WWS. However, Panicucci et al. reported that 18% of the *POMT2* variants linked to LGMDR14 were also found in more severe disorders, such as CMD and WWS, which suggested that predicting the phenotype based on only the genotype could be challenging [10].

Our ability to correlate the genotype with the phenotype was constrained by the abundance of compound heterozygous variants that vary in type and location [27]. After variants occur in the *POMT2* gene, there were two possible outcomes: (1) a total lack or partial deletion of the pomt2 protein and (2) the synthesis of a complete protein with a substitution or an insertion of amino acids [25]. Alterations in protein structure could have diverse effects on the functionality, and cause various diseases to develop. Therefore, investigating the impact of *POMT2* variants on the 3D protein structure was imperative for comprehending the clinical manifestations of diverse variants. In our analysis, the variants c.1006 + 1G > A, c.700_701insCT and c.170G > A produced truncated proteins that may impair the normal structure and function of pomt2. To better elucidate the genotype-phenotypic relationship of the three patients, we used molecular dynamics simulations to analyze the effects of truncated proteins on the function and stability. We conducted a 100-ns molecular dynamics simulation of three mutant proteins and the wild-type protein. MT1 and MT3 had larger RMSD values than WT, which indicated a decrease in protein stability. The RMSF results revealed that MT1 and MT2 had different degrees of influence on the N-terminal stability of the pomt2 protein (Fig. 4). The decreased SASA and Rg values for three mutants indicated reduced flexibility and potential interaction impairment (Figure S4). Moreover, the three variants affected the number of hydrogen bonds and their major secondary structures. These changes may affect the binding ability and catalytic function of the protein with other molecules, which affected the O-mannoylation of α-DG. This method well simulated the 3D helical structure of the three mutants and the wild-type protein, and used the variation information to monitor the changes of the trimer structures. Molecular dynamics simulations could help establish a link between genotype and phenotype to evaluate their complexity, but this approach has not been applied in previous *POMT2*-related studies.

## Conclusions

In summary, this study provided a comprehensive analysis of three Chinese patients with adult-onset LGMDR14, including their clinical features, muscle pathology and molecular genetics. We identified three novel variants: c.700_701insCT, c.812 C > T and c.170G > A. The abnormal mRNA processing and splicing mechanism caused by the intron variant c.1006 + 1G > A were elucidated. Additionally, we analyzed the NMD effects of the c.1006 + 1G > A and c.700_701insCT variants at the transcriptional level by pyrosequencing and qPCR. Furthermore, we used molecular dynamics simulations to investigate the structural and functional changes in the mutant proteins. Integrating clinical findings with mRNA processing analysis and bioinformatic data from molecular dynamics may help us objectively assess the impact of genetic variants on phenotypic manifestations. Ultimately, our study extended the phenotypic and genetic spectrum of *POMT2*-associated LGMDR14 and provided valuable insights into subsequent accurate diagnosis and potential treatment strategies for alpha-dystroglycanopathies.

## Electronic supplementary material

Below is the link to the electronic supplementary material.

Supplementary Material 1

## Data Availability

Data available on request from the authors. All data generated in this study was included in this published article and its supplementary files.

## Abbreviations

POMT2: The gene encoding Protein O-mannosyltransferase 2
pomt2: Protein O-mannosyltransferase 2
a-DG: α-dystroglycan
CK: Serum creatine kinase
ACMG: American College of Medical Genetics and Genomics
RNA: Ribonucleic acid
mRNA: Messenger RNA
NMD: Nonsense-mediated mRNA decay
MRI: Magnetic resonance imaging
LGMDR14: Limb girdle muscular dystrophy R14
α-DGP: Alpha-dystroglycanopathies
WWS: Walker-Warburg syndrome
CMD: Congenital muscular dystrophy
MD: Molecular dynamics
HGMD: Human gene mutation database
DNA: Deoxyribonucleic acid
cDNA: Complementary DNA
MRC: Medical research council
NGS: Next-generation sequencing
PCR: Polymerase chain reaction
RT-PCR: Reverse transcription PCR
qPCR: quantitative PCR
EMG: Electromyography
RMSD: Root mean square deviation
RMSF: Root mean square fluctuation
SASA: Solvent-accessible surface area
Rg: Radius of gyration
