# Supplementary material for: Novel POMT2 variants associated with limb-girdle muscular dystrophy R14: genetic, histological and functional studies
Source: Orphanet J Rare Dis. 2025 Mar 3;20:99. doi: 10.1186/s13023-025-03578-7 (PMC11921505; doi:10.1186/s13023-025-03578-7)
Supplement: Supplementary file 1 — Supplementary Material 1 [file 13023_2025_3578_MOESM1_ESM.docx]

**Novel *POMT2* variants associated with limb-girdle muscular dystrophy R14: genetic, histological and functional studies.**

Guiguan Yang^1,*^, Xiaoqing Lv^1,*^, Wenjing Wu^1^, Guangyu Wang^1^, Mengqi Yang^1^, Yifei Feng^1^, Chuanzhu Yan^1^, Meirong Liu^2†^, Pengfei Lin^1†^

^1^Department of Neurology, Shandong Key Laboratory of Mitochondrial Medicine and Rare Diseases, Research Institute of Neuromuscular and Neurodegenerative Diseases, Qilu Hospital of Shandong University, Jinan, Shandong, China.

^2^Institute of Stroke Research, Soochow University, 188 Shizi Street, Suzhou 215006, Jiangsu Province, China; Department of Neurology, The First Affiliated Hospital of Soochow University, 188 Shizi Street, Suzhou 215006, Jiangsu Province, China.

*These authors contributed equally to the work.

^†^**To whom correspondence should be addressed:**

Pengfei Lin, Department of Neurology and Research Institute of Neuromuscular and Neurodegenerative Diseases, Qilu Hospital of Shandong University, Jinan, Shandong 250012, China. Email: [lpfsdu@foxmail.com](mailto:lpfsdu@foxmail.com)

Meirong Liu, Department of Neurology, The First Affiliated Hospital of Soochow University, 188 Shizi Street, Suzhou 215006, Jiangsu Province, China. Email: [meizai26@163.com](mailto:meizai26@163.com)

**Supplementary materials and methods**

**Quantitative PCR assay**

RNA extraction was conducted utilizing the FastPure Cell/Tissue Total RNA Isolation Kit from Vazyme. The extracted RNA was then converted to cDNA through reverse transcription, employing HiScript III RT SuperMix for qPCR (+gDNA wiper), also from Vazyme, following the manufacturer's guidelines. Quantitative PCR analysis was carried out on QuantStudio 3 and 5 Real-Time PCR Systems (Thermo Fisher Scientific) using ChamQ Universal SYBR qPCR Master Mix (Vazyme). The sequences of primers used in this study can be found in the supplementary materials provided online. Primers were designed to detect whether nonsensemediated mRNA decay was triggered in the patient 1 and patient 2. Primer 1 was designed to detect the total *POMT2* mRNA level in the patient1 and the control. Primer 2 was designed to detect the total *POMT2* mRNA level in the patient 2 and the control.

**Primers utilized in this study**

1. **Primer sequences used for Sanger sequencing.**

|  | **Forward (5’-3’)** | **Reverse (5’-3’)** |
| --- | --- | --- |
| **c.1006+1G>A** | AGCGCTCCTGGGAAATCAC | CAACACTCAGAAAGCGGTGC |
| **c.295C>T** | CAGTGGCCAGCTTGATGTCT | CTGAGCCCAGAGGACCCTTA |
| **c.1261C>T** | CCCCTCTCATCCTCCACAGATG | AGGCCCATAACTTTTACGTCCT |
| **c.700_701insCT** | TCACTTGTGAAGAAGGAGCCC | TACTCAGCAACATCAGGGAGC |
| **c.812C>T** | CTCCAGCCTGGGTAACAGAG | TGCCAAATTGAACTCCGTCT |
| **c.170G>A** | AACCCACCCGATACCTCAA | GAGCTGCCCTCTTGTGACTC |

1. **Primer sequences used for RT‒PCR analysis**

|  | **Forward (5’-3’)** | **Reverse (5’-3’)** |
| --- | --- | --- |
| **Prime 1** | CTGTCCTTCGCCACCCGCT | AGAATCCTCTCATTCCCAT |
| **Prime 2** | CGCACATCTGTTGGGATGAG | CTCAGGTAGCCAGCAAGACC |

**Molecular Dynamics Simulation Parameters and Statistical Analysis**

Molecular dynamics (MD) simulations were performed to investigate the structural dynamics and stability of both the wild-type (WT) and mutant proteins. Relevant parameters, including root-mean-square deviation (RMSD), root-mean-square fluctuation (RMSF), solvent-accessible surface area (SASA), radius of gyration (Rg), and the number of hydrogen bonds (Hbonds) and major secondary structure elements, were extracted from the simulation trajectories. To compare the differences between the WT and mutant, we used Kernel Density Estimation (KDE), a non-parametric statistical method, to analyze the distributions of these parameters. KDE was performed using Python 3.8 with scipy 1.7.0, applying Gaussian kernels with bandwidths determined by Scott's rule. Resulting density plots were generated using matplotlib 3.4.2, displaying the parameter values on the x-axis and normalized density on the y-axis. WT and mutant distributions were represented by different colors for direct comparison. Statistical significance of distribution differences was assessed using the Kolmogorov-Smirnov test. KDE estimates the probability density function of a random variable by smoothing the observed data points with a kernel function, effectively capturing the underlying distribution without assuming a specific parametric form.

**Supplementary results**


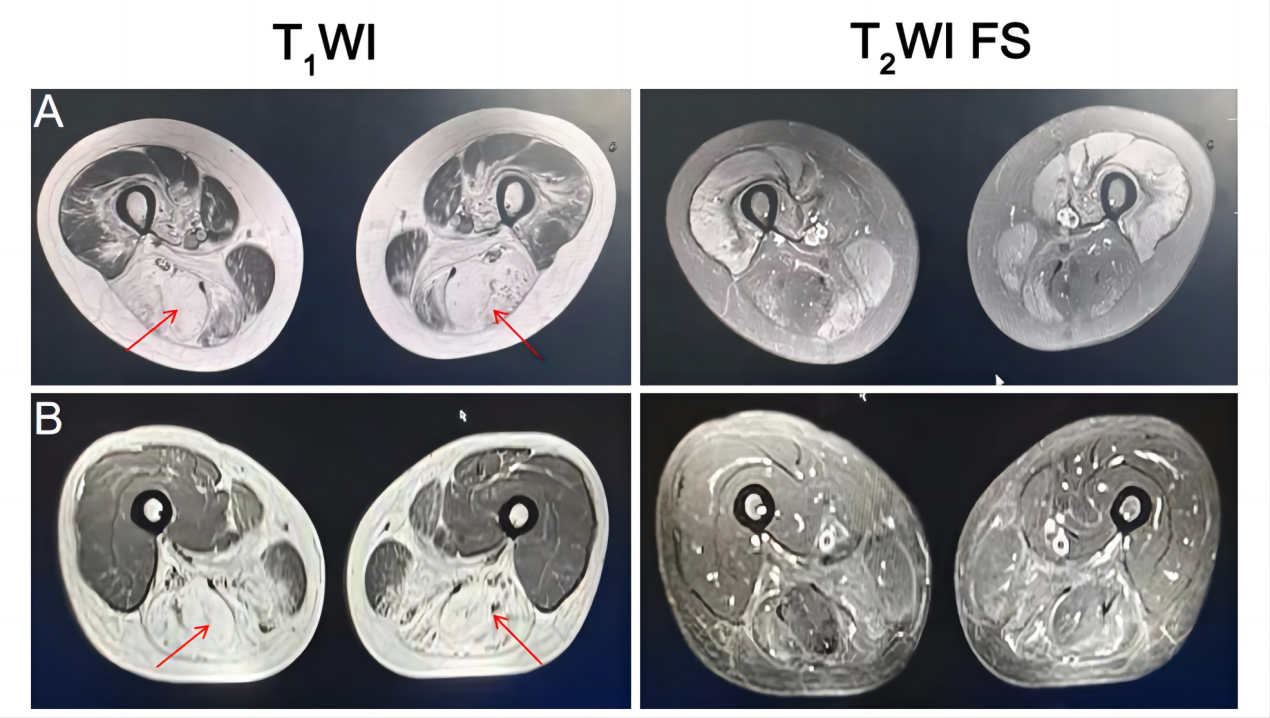


**Figure S1. Muscle MRI results of patient 1 and patient 3.**

**(A)** For patient 1, fatty infiltration was mainly present in bilateral posterior thigh (red arrows). (**B)** For patient 3, fatty infiltration was mainly present in bilateral posterior thigh (red arrows). FS: Fat Suppression


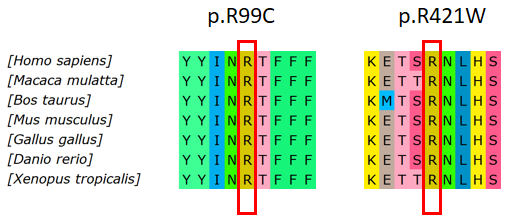


**Figure S2.** Multiple sequence alignment for the identified variant (c.295C>T, p.R99C) and (c.1261C>T, p.R421W).


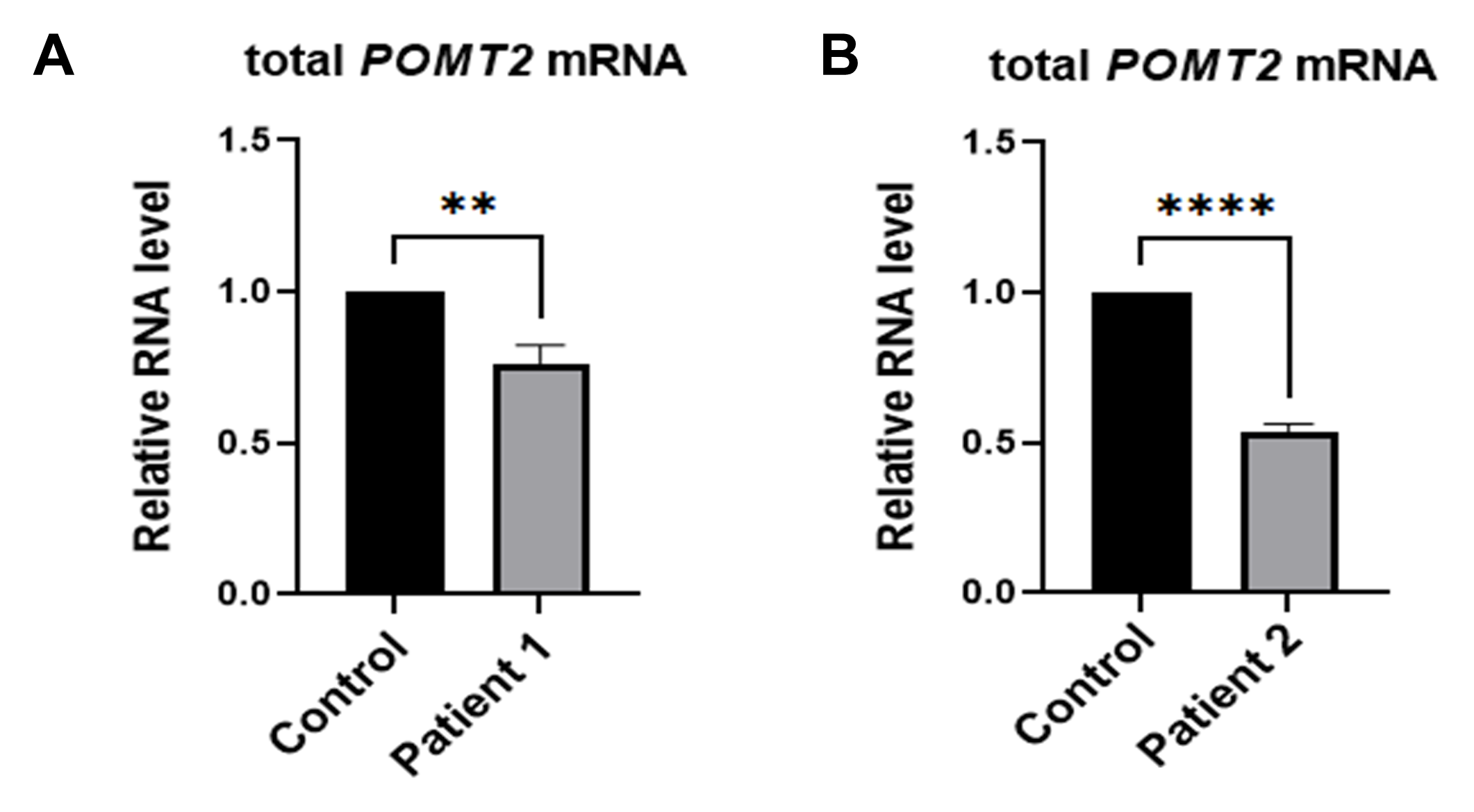


**Figure S3.** **A** Quantitative PCR assay showed 22% of the *POMT2* mRNA was degraded in the quadriceps femoris of the patient 1 compared to the control. **B** Quantitative PCR assay showed 46% of the *POMT2* mRNA was degraded in the quadriceps femoris of the patient 2 compared to the control. An unpaired t test was used to compare the patient and control. *p* < 0.01 (**), *p* < 0.0001(****).





**Figure S4. Molecular dynamics simulation statistical results.**

**(A-B)** Time evolution of **(A)** radius of gyration (Rg) and **(B)** solvent accessible surface area (SASA) for wild-type and mutant proteins over 100 ns simulations. **(C-I)** Kernel density statistical plots illustrating the distributions of different parameters: **(C)** Root mean square deviation (RMSD), **(D)** Hydrogen bonds (H-bonds), **(E)** SASA, **(F)** Rg, **(G)** Coil, **(H)** α-Helix, and **(I)** β-Sheet content across the different constructs. The color scheme is consistent throughout, with Wild Type shown in blue, p.V234Afs*8 in red, p. L337Efs*15 in green, and p.W57* in purple.
